# ZnO Decorated Graphene-Based NFC Tag for Personal NO_2_ Exposure Monitoring during a Workday

**DOI:** 10.3390/s24051431

**Published:** 2024-02-22

**Authors:** Alejandro Santos-Betancourt, José Carlos Santos-Ceballos, Mohamed Ayoub Alouani, Shuja Bashir Malik, Alfonso Romero, José Luis Ramírez, Xavier Vilanova, Eduard Llobet

**Affiliations:** Universitat Rovira i Virgili, Microsystems Nanotechnologies for Chemical Analysis (MINOS), Departament d’Enginyeria Electronica, Països Catalans, 26, 43007 Tarragona, Catalunya, Spain; alejandro.santos@urv.cat (A.S.-B.); josecarlos.santos@urv.cat (J.C.S.-C.); mohamedayoub.alouani@urv.cat (M.A.A.); shujabashir.malik@urv.cat (S.B.M.); alfonsojose.romero@urv.cat (A.R.); joseluis.ramirez@urv.cat (J.L.R.); eduard.llobet@urv.cat (E.L.)

**Keywords:** wearable NFC tag system, NO_2_ gas sensor, working-day, metal oxide, graphene

## Abstract

This paper presents the integration of a sensing layer over interdigitated electrodes and an electronic circuit on the same flexible printed circuit board. This integration provides an effective technique to use this design as a wearable gas measuring system in a target application, exhibiting high performance, low power consumption, and being lightweight for on-site monitoring. The wearable system proves the concept of using an NFC tag combined with a chemoresistive gas sensor as a cumulative gas sensor, having the possibility of holding the data for a working day, and completely capturing the exposure of a person to NO_2_ concentrations. Three different types of sensors were tested, depositing the sensing layers on gold electrodes over Kapton substrate: bare graphene, graphene decorated with 5 wt.% zinc oxide nanoflowers, or nanopillars. The deposited layers were characterized using FESEM, EDX, XRD, and Raman spectroscopy to determine their crystalline structure, morphological and chemical compositions. The gas sensing performance of the sensors was analyzed against NO_2_ (dry and humid conditions) and other interfering species (dry conditions) to check their sensitivity and selectivity. The resultant-built wearable NFC tag system accumulates the data in a non-volatile memory every minute and has an average low power consumption of 24.9 µW in dynamic operation. Also, it can be easily attached to a work vest.

## 1. Introduction

Nitrogen dioxide (NO_2_) is a highly toxic gas that is found in outdoor air pollution but can also be found in indoor scenarios such as renovation or construction activities when using certain paints, adhesives or solvents, smoking areas, parking garages, and industrial facilities, among others [1]. NO_2_ is known to harm the human respiratory system. This pollutant gas can irritate airways and provoke respiratory diseases such as asthma, pharyngitis, and bronchitis [2,3]. Particularly, a lot of work positions around the world have a risk of exposing workers to this toxic gas, and studies have been conducted on this topic. U. Carbone et al. [4] concluded that occupational exposure to NO_2_ emissions in power plants is significantly associated with lung function abnormalities. N. Plato et al. [5] conducted a study to detect particles and NO_2_ exposure in workers inside the underground train system in Stockholm and classified the workers according to the level of exposure. Salonen H. et al. [6] determined that workers in places like schools and indoor offices have a risk of exposure to NO_2,_ mostly in the winter season. Dahmann D. et al., and Kurnia J.C. et al., agreed that workers from underground mining and tunneling need to give special attention to the level of pollutants in the atmosphere, especially NO_2_ [7,8]. According to the European Chemical Agency (ECHA), the Occupational Exposure Limits (OELs) in a Long-Term Period Limit (LTEL) are equal to 0.5 ppm, and the OELs in a Short-Term Period Limit (STEL) are equal to 1 ppm [9]. Additionally, this contaminant gas is also dangerous to the global ecosystem and the environment, since it has adverse effects on water, soil, and the atmosphere [10]. Therefore, monitoring NO_2_ is a crucial issue for environmental and human well-being, and numerous types of sensors have emerged in recent years [11,12].

Chemoresistive sensors have become an attractive gas monitoring option due to their easy fabrication, miniaturization, and low production cost [13,14]. They consist of a sensitive layer placed between two electrodes, which enables measuring the variation in electrical conductivity when reacting to specific gases [15]. Some of the most widely known and used nanomaterials for the growth of the active layer are metal oxides (MOXs) [16]. MOXs have some advantages, like their small size, low manufacturing cost, simple read-out-chain design, and short response time when reacting to a chemical analyte [17,18]. But also, despite being useful and effective for a long period, they have several disadvantages, such as poor selectivity, baseline drift, high sensitivity to humidity, and high power consumption since they usually work at high temperatures [19,20]. In recent years, graphene-based materials have risen due to their excellent sensing properties [21,22]. The combination of graphene with different MOXs has also been studied to increase the selectivity, improve the baseline recovery, shorten the recovery time, and decrease the power consumption as they could work at room temperature [23,24]. Table S1 in the Supplementary Materials, presents a comparison of graphene-based gas sensors for NO_2_ detection at room temperature. To prove the feasibility of the wearable NFC tag system developed during this work, graphene decorated with 5 wt.% zinc oxide (ZnO) was used due to the previous experience of our research group working with a combination of these materials [25]. The authors compared different wt.% ZnO loading on a graphene-based sensor and concluded that the 5 wt.% loading performs better than higher and lower loadings according to sensing capabilities toward NO_2_ concentrations.

Since wearable devices must have specific features such as lightweight, wireless connectivity, and low power consumption, building a gas sensor as a wearable device is a substantial challenge [26,27,28]. Nonetheless, plenty of wearable gas sensors and healthcare wearable systems have been recently developed [29]. Chen et al., demonstrated a Bluetooth wearable NO_2_ sensor based on zinc sulfide nanoparticles/nitrogen-doped reduced graphene oxide [30]. Also, a flexible gas early warning module was proposed and designed by Zhang et al., based on flexible electronic technology [31]. Likewise, a highly flexible epidermal design and clinical implementation of a novel ECG and heart-rate logging wearable sensor were presented by Lee et al. [32]. Moreover, Lin et al. [33] reported the integration of NFC functionality into textiles and demonstrated continuous physiological monitoring of the spinal posture. All these wearable devices use several wireless communication technologies to send the data from sensors to the final user interface or a database for further processing. These wireless technologies employ Bluetooth, Ultra-High Frequency Radio Frequency Identification (UHF RFID), and Near Field Communication (NFC). They have several advantages and disadvantages, regardless of power consumption, communication range, and price [34,35]. In recent years, the integration of NFC technology with low-cost electronics and sensors has facilitated a variety of new sensing applications [35]. A study of a wearable carbon dioxide sensor was reported by Escobedo et al. [36], describing the performance of a wearable NFC tag to be used for non-invasive gas determination. Zhang et al. [37] reported a flexible NFC system based on an ammonia sensor using reduced graphene oxide decorated with silver nanoparticles, which sense low concentrations (5 ppm) at room temperature. Likewise, Escobedo et al. [38] presented a full-passive flexible multi-gas sensing tag for determining oxygen, carbon dioxide, ammonia, and relative humidity readable by a smartphone through NFC technology. Moreover, wireless, passive, flexible, and low-cost NFC tag sensors based on commercial NFC tags for biochemical sensing were demonstrated by Xu et al. [39]. Additionally, Salehnia F. et al. [40] discuss the development of a battery-free NFC sub-ppm gas sensor for distributed gas monitoring applications, using a laser-induced graphene (LIG) sensor for NO_2_ detection. The use of NFC technology in the aforementioned studies has advantages, such as the fact that the systems can be used without batteries, harvesting energy from the NFC field. Nevertheless, this is also a disadvantage since the systems cannot acquire historical data from the sensor for a long period of time. They only obtain the specific value of the sensor data when the NFC tag is near the NFC reader or when they have sufficient energy in the harvest-storage element. Table S2 in the Supplementary Materials, presents a comparison of the wearable NFC gas sensors on a flexible substrate.

To overcome this challenge, we demonstrate for the first time in this paper the fabrication of a wearable cumulative battery-operated gas sensor based on bare graphene (BG) decorated with 5 wt.% ZnO nanoflowers (NF) and nanopillars (NP) for on-site NO_2_ monitoring. The communication stage between the wearable system and the reader is conducted through NFC technology, and the cumulated data can be monitored on a smartphone (an NFC reader) using a custom-made test application. This wearable NFC tag system was built to prove the concept of being used in a target application in which human resources could be exposed to NO_2_ concentrations during working hours. This small wearable system is easy to integrate into a working vest. Using it, a worker could easily read the data from sensors with a smartphone and obtain the gas exposure for a whole working-day period. It is worth mentioning that the scalability of this system could be as high as the software solution running on the smartphone. Moreover, since this procedure could also be used with different sensing materials, we believe these results will provide a new perspective on wearables for the on-site monitoring, recording, and analysis of threatening gases.

## 2. Experimental

### 2.1. Wearable NFC Tag System Preparation

**Figure 1:**
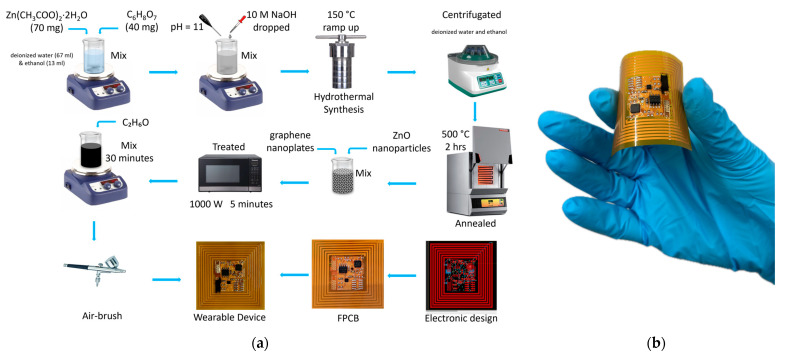
(**a**) Schematic description of the synthesis of zinc oxide nanoflowers and nanopillars, preparation of graphene ZnO composite, electronics design/assembling process, and air-brush process. (**b**) Picture of the wearable NFC tag system.

Figure 1a presents a description of the fabrication process of the wearable NFC tag system. First, the synthesis of the nanoparticles was performed. A hydrothermal method was used to synthesize ZnO nanoparticles. A total of 70 mg of zinc acetate dihydrate (Zn(CH_3_COO)_2_·2H_2_O) (Sigma Aldrich, St. Louis, MO, USA, CAS: 5970-45-6) and 40 mg of citric acid (C_6_H_8_O_7_) (Sigma Aldrich, CAS: 77-92-9) were dissolved in a mixture of deionized water (67 mL) and ethanol (13 mL) under vigorous stirring. A total of 10 M sodium hydroxide (NaOH) (Sigma Aldrich, CAS: 1310-73-2) was dropped into the stirring solution until the pH reached 11. The solution turns hazy white after the addition of NaOH. After 4 h of continuous stirring, the solution turns milky. The solution was then transferred to a 100 mL capacity autoclave with a Teflon liner. The autoclave was heated at 150 °C with a ramp-up temperature of 10 °C per minute for 20 h for NF synthesis and 17 h for NP synthesis. The autoclave was allowed to cool naturally to room temperature. The solution was centrifuged to obtain the white precipitate, thoroughly washed with deionized water and ethanol several times, and dried at 80 °C overnight. The nanomaterials were annealed at 500 °C for 2 h under a synthetic air environment in a muffle furnace (Carbolite CWF 1200, Carbolite Gero Ltd., Neuhausen, Germany) before mixing with graphene.

The next step was the preparation of the ZnO-graphene hybrid/composite using commercial graphene nanoplates (Strem Chemicals, Newburyport, MA, USA). The nanoplates were mixed with the previously prepared ZnO nanoparticles to make two different powders, one with 5 wt.% of NF and the second with 5 wt.% of NP. The mixing process was simple and low-cost, where the previously obtained powders were dissolved in ethanol (Scharlab, Sentmenat, Spain, CAS: 64-17-5) and put under magnetic stirring for 30 min. Later, the two different solutions were filtered using filter paper. Afterward, the mixed powders were treated under a microwave at a power of 1000 W for about 5 min. Solutions of 10 mL of Ethanol mixed with 1 mg of graphene powder (decorated or pristine) under magnetic stirring for half an hour were prepared to be airbrushed onto the gold electrodes. Simultaneously, a third step was focused on the electronic design using a free license for non-commercial use and evaluation purposes of Autodesk Eagle version 9.6.2. Then, the final flexible printed circuit board (FPCB) was fabricated (JLCPCB, Shenzhen, China). Afterward, components were populated on the board, and the programming and debugging of the firmware were performed using the MCUXpresso Integrated Development Environment v11.7.1_9221 from NXP Semiconductor. Finally, the solutions were airbrushed on top of the interdigitated electrodes (IDEs) using a DISMOER airbrush tool. Figure 1b shows the obtained wearable NFC tag system.

### 2.2. Electronic and Firmware Design

The FPCB was designed in an area equal to 45 × 45 mm^2^. It was divided into four main parts: First, an NFC-dedicated System on Chip (SoC) with an ARM Cortex-M0+ microcontroller controls the electronic board. Features such as an ultra-low-power Real Time Clock (RTC) and a 4 kB EEPROM memory were suitable to swap the system between power modes and to store samples from a 12-bit successive-approximation charge-redistribution analog to digital converter (ADC). Second, the rest of the electronics is integrated by a DC-DC converter, which aims for the proper stabilization of 3 V across the board, suppressing the voltage variation of the battery when current consumption changes. Additionally, the DC-DC converter has a shutdown feature permitting the possibility of hard-shutdowning the whole board. Also, the electronic is integrated into the analog front end with an operational amplifier and a resistor to bias the sensor. The third main part is a pair of IDEs to be coated with gas-sensitive layers. The IDEs have 5 pairs of fingers; the width and gap distance between them are equal to 300 μm, and the length is equal to 1.90 mm. Fourth, the NFC antenna was designed following the recommendations of a series of application notes and using the online NFC Antenna Design Tool from NXP Semiconductors [41]. As a result, the antenna dimensions: length and width were set to 43 mm, and the number of turns was set to 7. The copper antenna conductor has a width of 1 mm, a spacing of 0.5 mm, and a thickness of 0.1 µm. The substrate thickness is 0.11 µm, and the relative electrical permittivity of Kapton is equal to 3.2. The resultant antenna has an inductance value of 2.62 µH at 13.56 MHz.

**Figure 2:**
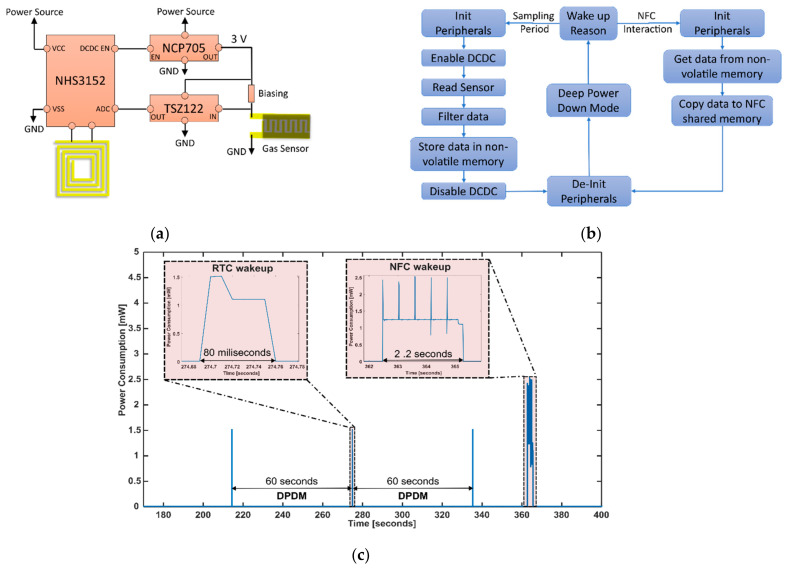
(**a**) Schematic design of the wearable NFC tag system. (**b**) Software interaction diagram. (**c**) Power consumption of the wearable NFC tag system during DPDM, wake-up process every 60 s to acquire the data from the sensor, and one NFC read interaction.

Figure 2a presents the schematic design of the board. The SoC NHS3152 from NXP Semiconductor runs custom-made software that controls the rest of the electronics. Once the board is powered on, the NHS makes sure that the DC-DC converter, NCP705, is turned off and goes to the Deep Power Down Mode (DPDM). This action ensures that all the hardware is off, except for the Power Management Unit (PMU) and the RTC. Also, all digital functional pins are tri-stated except for the reset pin. Before entering this mode, the wake-up sources of the SoC need to be correctly configured. In this design, there are two possible ways; Figure 2b shows the details. The first one is related to acquiring the data from the sensor. Using the RTC, a scheduled wake-up task is performed every 60 s. The SoC is woken up, initializes the necessary peripherals, and enables the DC-DC converter to power the analog channel. The channel is based on an operational amplifier, TSZ122, in a voltage follower configuration to avoid the current leak of the sensor when biased. In this manner, placing the rightful value of the bias resistance ensured a voltage variation that covered the full range of the ADC (0 V–1.6 V). Afterward, an analog-to-digital conversion is conducted, a conditioning of the signal is performed using a windowed filter, and the data is stored inside a non-volatile memory, EEPROM. Then, after de-initializing all the peripherals and disabling the DC-DC converter, the system goes to DPDM again. This procedure takes less than 80 ms, and the peak power consumption during the acquisition is about 1.5 mW, as shown in Figure 2c. It is important to notice that this fast reading-sensor procedure can be achieved because the sensor does not need a warm-up time to be read. The second way to wake up the board is related to NFC field detection. This event occurs every time an NFC reader reads the data. Subsequently, right after the initialization of the peripherals, the data is read from the EEPROM memory and retained in the NFC shared memory to be sent to the NFC reader. This time, the rest of the electronics are kept in off mode, so the average power consumption is lower (1.25 mW) than the wake-up process explained before but takes around 2 s. Finally, when the reader is not nearby, the board goes to DPDM again, and then the power consumption of the whole hardware is the lowest, around 3 µW. The power consumption test was performed using the Keysight B2902A Precision Source/Measure Unit (Figure S1 in the Supplementary Materials shows the details). The wearable NFC tag system shows an average power consumption of 24.9 µW, which is suitable to be used with low-capacity coin cell batteries and, therefore, low weight. (Ex: using a battery CR2032 (Capacity: 210 mAh), the system would theoretically work for more than one year and weigh less than 5 g).

### 2.3. Active Layer Characterization

The X-ray diffraction (XRD) measurement was performed using a D8-Discovery LYNXEYE-XE-T diffractometer, configured in conventional analytical conditions. The angular 2θ diffraction ranged from 15 to 80 degrees. The collection of the data was performed with an angular step of 0.05 degrees at 0.5 s per step and sample rotation. CuKα radiation was obtained from a copper X-ray tube operated at 40 kV and 40 mA. Divergence slit 0.5 mm, anti-scatter slit 5.13, primary and secondary Soller 2.5 degrees, detector opening 2.94 degrees, air-scatter in automatic configuration, and default program settings. Also, a Field Emission Scanning Electron Microscope (FESEM) Scios 2 DualBeam was used to study surface morphology and check the NP and NF distributions on the graphene layer. Sample characterization was performed at a high vacuum, and the electron acceleration voltage was established between 2 and 5 kV. The energy-dispersive X-ray (EDX) incorporated in the FESEM Scios was used to check the chemical composition of the active layer. A Raman spectrometer (Renishaw, plc., Wotton-under-Edge, UK) utilizing a coupled confocal microscope (Leica DM2500 Microsystems, Leica Microsystems GmbH, Wetzlar, Germany) with a laser wavelength of 785 nm was used to check the structural fingerprint by vibrational modes of the molecules. To determine the surface area of the nanomaterials, the Brunauer–Emmett–Teller (BET) nitrogen adsorption–desorption isotherms of the ZnO nanostructures were measured from the Quantachrome Instrument: QuadraSorb Station 3 (Quantachrome Instrument, Boynton Beach, FL, USA). Samples were outgassed at 100 °C for 5 h, and the bath temperature was 77.3 K.

### 2.4. Gas Measurement System

**Figure 3:**
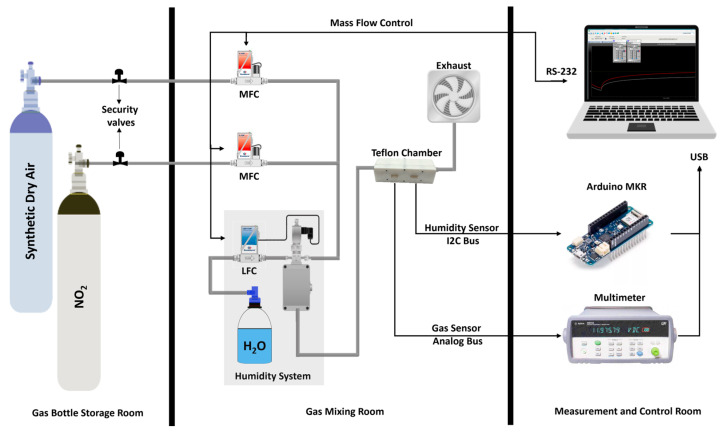
Schematic of the gas measurement system.

The gas-sensing characteristics of the materials were determined using the system illustrated in Figure 3. The gas measurement system consists of different gas bottles with calibrated gas concentrations balanced in dry air, a carrier gas with zero-grade dry air, and an airtight Teflon^®^ chamber with an inner volume of 25.85 cm^3^. The gases were delivered into the chamber through a computer-controlled mass-flow system to ensure the reproducibility of the concentrations and a constant low flow of 100 mL/min. The mass-flows are controlled by software named Flow Plot v3.34, running on a Windows-based computer. To fix the sensors inside the chamber, the electrodes were separated from the rest of the electronics using a cutter. Afterwards, the three different materials were air-brushed as explained in Section 2.1 and carefully soldered using an SMD4300AX10 solder paste to a support that fits into the chamber connector. In that manner, the three gas sensors (one of each material: NP, NF, and BG) were put inside the chamber and left under dry synthetic air for a long period to have a stable baseline resistance. Pulses of NO_2_ gas were injected into the chamber with different concentrations ranging from 50 ppb to 1 ppm, as follows: 50 ppb, 250 ppb, 500 ppb, 750 ppb, and 1 ppm; dry air was supplied in between the gas pulses to recover the baseline and keep the constant flow. All the measurements were performed at room temperature, and the gas exposure time was 35 min and the recovery dry airtime was 70 min for each pulse. This cycle of pulses was repeated 4 times, with 2 h of recovery time between cycles. The sensor resistance was measured and stored employing an Agilent 34972A multimeter (Keysight, Santa Rosa, CA, USA) and software from Agilent Technologies Bench Link Datalogger 3 running on the same computer mentioned before.

The humidity effect on the sensing performance was evaluated by humidifying (at room temperature) the gas stream through a controller evaporator mixer from Bronkhorst (Bronkhorst High-Tech, Ruurlo, The Netherlands). The percentage of humidity and the temperature during the measurements were checked using a digital sensor SENSIRION SHT85 (SENSIRION, Stäfa, Switzerland) placed inside the chamber. The accuracy of the SHT sensor is ±1.5% RH and 0.1 °C. An Arduino-based board was used to bridge the SHT sensor and the computer, interfacing the I^2^C bus and USB. Similarly, to check the selectivity, the sensors were exposed to benzene, toluene, carbon monoxide, ethanol, ammonia, and hydrogen. The exposures were performed using several pulses of the same level of concentration according to the STEL [4] of each gas or the maximum capacity of the available bottle in the storage room. For benzene was 1 ppm (STEL: 1 ppm), toluene was used the maximum capacity of the bottle 10 ppm (STEL: 50 ppm), carbon monoxide: 20 ppm (STEL: 20 ppm), ethanol was the maximum capacity of the bottle 20 ppm (STEL: 1000 ppm), ammonia was 50 ppm (STEL: 50 ppm) and hydrogen was the maximum capacity of the bottle 1000 ppm. In all cases, the sensing responses were calculated using Equation (1):

(1) $$Response\;\left[\%\right]=\frac{\left(R_{c}-R_{b}\right)}{R_{b}}\times 100\%$$

where *R_c_* is the value of the resistance of the sensor before being exposed to a cycle of pulse train of the target gas and *R_b_* corresponds to the value of the resistance after the target gas exposure in each pulse.

## 3. Results and Discussion

### 3.1. Active Layer Characterization

**Figure 4:**
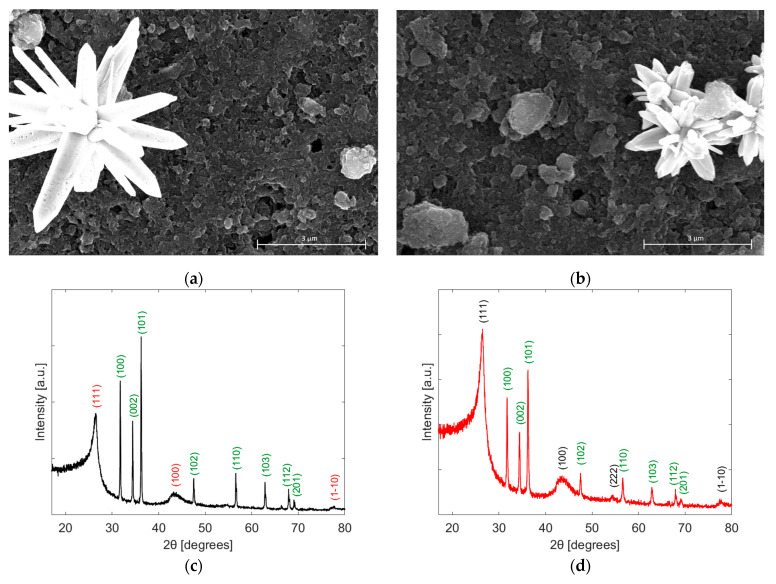

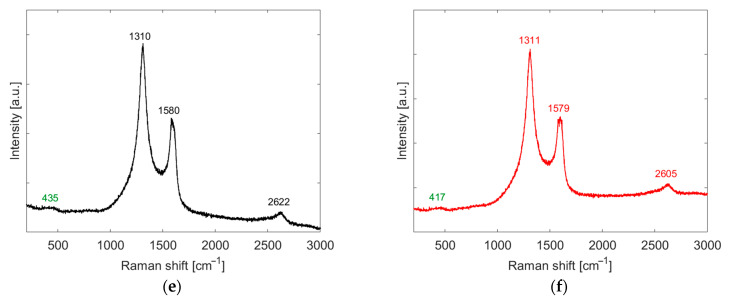
FESEM images using Back-scattered electron detector of (**a**) NP, (**b**) NF; XRD diffractograms of (**c**) NP, (**d**) NF; Raman spectra of (**e**) NP, (**f**) NF.

Figure 4a,b present the obtained FESEM images of NP and NF, respectively. A secondary electron detector was used to obtain images on a grayscale, with the nanoparticles as bright materials and a dark background representing the graphene layer. The images were taken on a 3 µm scale. Figure 4a shows the nanopillar structure of the ZnO with some porous formation (small dots) on the surface of the nanopillars, which increases the sensing performance of the active layer. These defects are incorporated into the material by microwave treatment. Figure 4b shows the morphology of the ZnO in a nanoflower shape, in the middle of the graphene layers. The FESEM image of BG is presented in Figure S2 in the Supplementary Materials.

The BET surface areas of the ZnO nanoparticles used for decorating the NP and NF sensors were measured by the nitrogen adsorption–desorption process. The surface area of the NF (2.499 m^2^ g^−1^) was found to be smaller, yet close to the surface area of the NP (2.990 m^2^ g^−1^).

A quantitative elemental analysis using EDX can be found in the Supplementary Materials, Figure S3 for the NF. The spectrum shows different peaks such as oxygen, carbon, and zinc present on the surface. The strongest peak of zinc in the EDX spectrum is explained by the image being taken right on top of one of the nanoflowers on the surface. An equivalent spectrum can be found when analyzing the NP, Supplementary Materials Figures S4 and S5 for BG.

Figure 4c,d show the XRD diffractograms for NP and NF materials, respectively. In this case, the samples were tested in powder form; therefore, a clear spectrum of the materials was obtained. It is noticed that the reflection peaks confirm the presence of carbon shown in the indices (111), (100), and (1–10) for the NP diffractogram. Also, for the NF diffractogram, it is noticed that there is an additional indicator of carbon with quite a low intensity (222).

Figure 4e,f show the Raman spectra of the NP and NF samples, respectively. D-band is located at 1310 and 1311 cm^−1^ in each case, which indicates the presence of defects in the graphene lattice. The G-band located at 1580 and 1579 cm^−1^ reveals the stretching of C-C bonds in both sensors. Moreover, the 2D-band is located at 2622 and 2605 cm^−1^, and its intensity could be an indicator of the number of graphene layers and is lower than the previous bands. Also, it is observed in both sensors a small peak at 435 and 417 cm^−1^, which can be attributed to the presence of ZnO in the active layer, normally at 430 cm^−1^. The Raman spectrum of BG is shown in Figure S6 in the Supplementary Materials.

### 3.2. Gas Sensing Results

**Figure 5:**
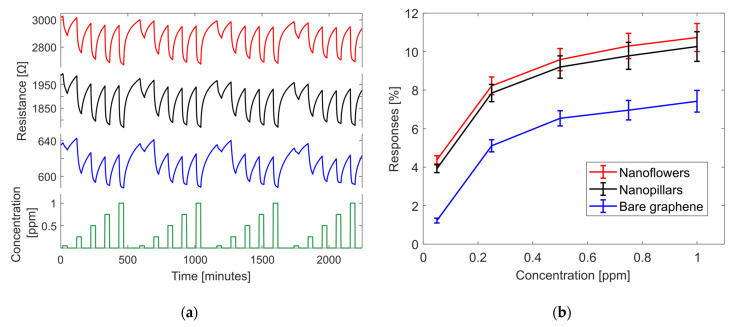
(**a**) Repeatability of the measurement in dry air conditions, four cycles of pulses of 50, 250, 500, 750 ppb, and 1 ppm of NO_2_ concentration (green color) and resistance variation of graphene-based sensors decorated to NF (red color), NP (black color) and BG (blue color). (**b**) Comparison between the calibration curves of NF, NP, and BG in dry air conditions.

Figure 5a shows the dynamic performance of the sensors under NO_2_ exposure. The three materials have high baseline stability and good repeatability over the whole range of the measurements. It can be noticed that the electrical conductivity of the sensing layer increases due to the normal behavior of a p-type material such as graphene reacting to an oxidant gas such as NO_2_. The signal-to-noise ratio is high since the sensors have a stable waveform variation of the resistance value during the sharp and fast waveform changes, right after the gas gets inside the chamber. Furthermore, it is noticed that the response of the sensor increases while the target gas concentration increases. Figure 5b shows the comparison between the calibration curves of the responses of the three sensors. Doped sensors have a bigger response than BG, and NF shows itself as the best sensor. For higher levels of nitrogen dioxide concentrations (1 ppm), the average ratio between the NF and BG responses is 1.45. For the lowest level of concentration (50 ppb), the average ratio increases to 3.58, which means that the response of NF is around 3.5 times higher than for BG. The same trend can be found when analyzing the ratio between NP and BG. In that manner, sensors doped with NP and NF were chosen to continue this study.

**Figure 6:**
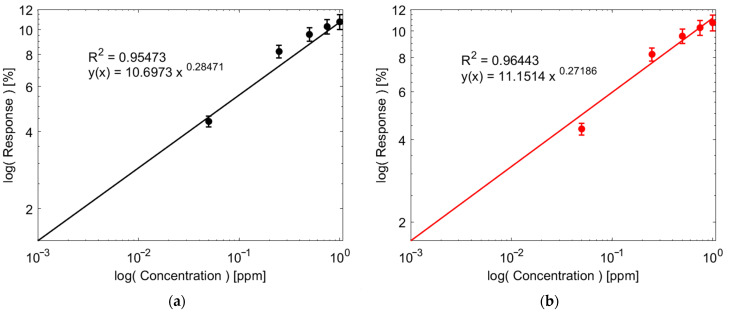

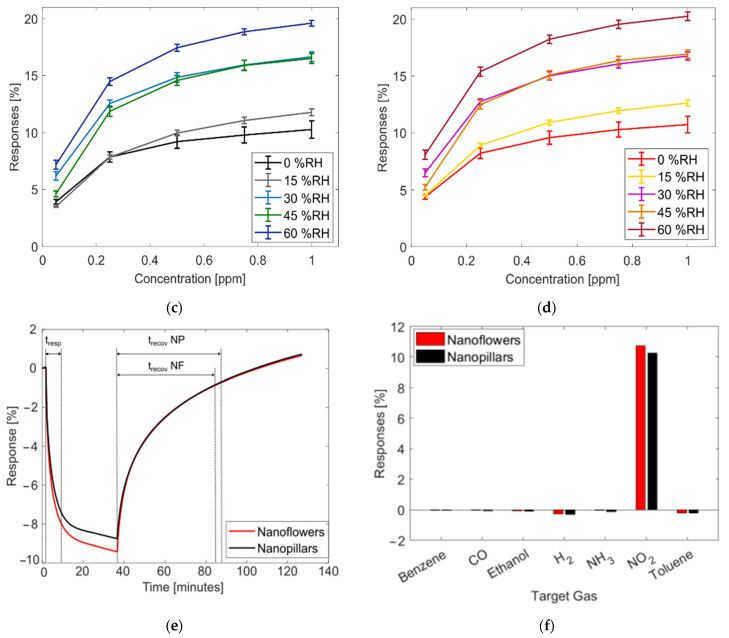
Fitted calibration curves for (**a**) NP, (**b**) NF; Response under different levels of humidity: 0%, 15%, 30%, 45%, and 60% RH for (**c**) NP, (**d**) NF; (**e**) Comparison of response and recovery time between NP and NF; (**f**) Comparison between responses of the sensors when exposed to different gases/vapors.

An extended comparison was performed between NP and NF. Figure 6a,b show the fitted calibration curves in log scale using a power-law model for both sensors, which have an R^2^ of 0.95 and 0.96 for NP and NF, respectively. As a result, the sensitivities of the two sensors at 1 ppm are equal to 3% ppm^−1^. Likewise, the theoretical Limit of Detection (LoD) was calculated using Equation (2).

(2) $$LoD=\frac{3.3\sigma }{S}$$

where *σ* is the standard deviation of the baseline resistance during a long period of measurement and *S* is the Sensitivity of the sensors. In each case, the NP sample has a theoretical LoD of 18.7 ppb and the NF sample of 13.3 ppb.

Since it is extensively reported that humidity affects the response of graphene-based sensors [42,43,44,45], similar measurements and analyses were performed under different relative humidity levels. Figure 6c,d present a comparison of the calibration curves for NP and NF in dry air conditions: 15% RH, 30% RH, 45% RH, and 60% RH. It was noticed that the baseline of the sensors increased after each increase in % RH. Also, the response of the sensors increases while the % RH increases. The Sensor doped with NF almost doubled its response in the total range of humidity variation. Likewise, this response increase has a clear advantage for the final application of this work due to real measurements being under humidity conditions, but it also has a disadvantage, which is the dependability of the sensor response to the variation of % RH. This issue is easy to address by including a humidity sensor in the electronic design and performing a multivariate analysis in the software solution. These two final ideas are out of the scope of this paper.

Furthermore, the response and recovery times of the sensors were evaluated in dry air conditions. Under 1 ppm of NO_2_ exposure, the sensors have a response/recovery time of around 10/47 and 10/49 min for NF and NP, respectively. Figure 6e shows the details of the comparison, and the response time is defined as the time taken to reach 90% of the full response after the introduction of the target gas. The recovery time is defined as the time taken to return to 90% of the baseline resistance after the flow of the target gas is stopped. They were calculated following Equations (3) and (4).

(3) $$Response\_T\;[sec]={Time}_{R\_90\%\_response}-{Time}_{R\_baseline}$$

(4) $$Recovery_{T}\left[sec\right]={Time}_{R_{90}{\%}_{baseline}}-{Time}_{R_{reacting}}$$

where ${Time}_{R_{90}\%\_response}$ is the time when the resistance value reaches 90% of the response after reacting to the target gas. The ${Time}_{R\_baseline}$ is the time before the sensor is exposed to the target gas. ${Time}_{R\_reacting}$ is the time before the sensor is exposed to dry air for a given recovery time and ${Time}_{R\_90\%\_baseline}$ is the time when the resistance value reaches the 90% of the previous baseline.

Finally, the sensors were exposed to different analytes to perform a selectivity comparison. To demonstrate that the wearable NFC tag system can be used in environments or industrial processes in which a combination of these analytes could be present in the atmosphere, the test was performed using VOC such as benzene, toluene, and ethanol [4,46,47,48], carbon monoxide [7,8,49], ammonia [8,50], and hydrogen [49]. Figure 6f presents a bar chart showing that the response of NP and NF to all these gases is less than 1% in dry air conditions. Therefore, the use of these two materials is suitable for sensing NO_2_ concentrations, even in the presence of other compounds.

### 3.3. Sensing Mechanism to NO_2_

The behavior of the sensors described in previous sections, regardless of their good selectivity towards NO_2_ concentrations and the increase in their responses as % RH increases, is consistent with the sensing mechanism of graphene-based sensors [45]. Graphene is a mild p-type nanomaterial that has a large surface area. This large area facilitates the adsorption of gas molecules, leading to rapid changes in the local carrier concentration and its conductivity. In the presence of oxidant gases, its conductivity is increased. Conversely, when detecting reducing gases, the conductivity decreases. Therefore, in the sensors used in this work, when reacting to NO_2_, the resistance value decreases, and while reacting to other gases such as benzene or hydrogen, the resistance value of the sensor increases. Considering p-type graphene loaded with n-type ZnO nanoparticles, the probable outcome is the formation of a p-n junction on the contact surface. This formation creates a depletion layer at the interface, with electrons flowing from the ZnO conduction band to the graphene. As a result, NP and NF sensors exhibit a higher resistance baseline compared to the BG sensor due to the injection of electrons from the n-type ZnO to the graphene, reducing the concentration of majority carriers in the p-type graphene and lowering the conductivity of the sensing film. When NP and NF are exposed to dry air, they interact with oxygen molecules, capturing electrons from the valence band and causing them to be adsorbed into the sensor surface (Equation (5)). When NO_2_ interacts with the sensor surface, two reactions may occur. First, NO_2_ can be adsorbed onto the hybrid nanocomposite (Equation (6)). Second, NO_2_ may interact with adsorbed oxygen species, leading to changes in the resistivity of the active layer (Equation (7)). In both cases, NO_2_ adsorption captures free electrons from the sensitive film, increasing the conductivity.

(5) $$O_{2}\left(g\right)+2e^{-}\to {2O}_{2{\left(ads.\right)}^{-}}$$

(6) $${NO}_{2}\left(g\right)+e^{-}\to {NO}_{2{\left(ads.\right)}^{-}}$$

(7) $${2NO}_{2\left(ads.\right)}+{O_{2-}+e}^{-}\to {2NO}_{3}^{-}$$

The p–n junction enhances the sensor sensitivity by expanding the depletion region when the film interacts with NO_2_ molecules. NP and NF exhibit higher sensing performance than BG. Likewise, NF has a better response to NO_2_ than NP, possibly because its contact area with the target gas is larger than that of NP. Finally, the presence of humidity enhances the sensitivity of graphene-based sensors. At room temperature, water molecules act as a mediated adsorption site for NO_2_, and additional electrons are released in the conduction band of the metal oxide material, leading to an increased response to the target gas [45].

### 3.4. Gas Measurement Using NFC Tag System

Using the built-in wearable NFC tag system, a gas measurement test was performed. The system, with airbrushed graphene decorated with NF material, was placed inside a chamber. In this manner, the volume of the environment in which the system was placed increased. A new acrylic chamber was used with a volume of 5000 cm^3^, and the system was powered using a coin cell battery, CR2032. Afterward, the system was left in synthetic dry air for one hour; in between, a stream of NO_2_ concentration of 1 ppm was supplied to the chamber for 15 min (constant flow of 100 mL/min). Figure S7a in the Supplementary Materials shows a picture taken of the test bench for this test.

To recreate different possible scenarios of exposure to nitrogen dioxide, two additional tests were performed as follows. First, NO_2_ at 1 ppm was supplied to the chamber for 10 min, followed by 10 min of dry air, and finally 500 ppb of NO_2_ for another 10 min. After recovering the sensor in dry air, a second test comprised supplying the chamber with 500 ppb of NO_2_ for 10 min, followed by a 10 min recovery in dry air, and ending with a 10 min exposure to 1 ppm of NO_2_. Figure S7b,c in the Supplementary Materials shows a screenshot of a smartphone of the acquired data in each test, respectively. It is seen in both cases that the NFC tag system can successfully obtain the variation in the resistance value under different levels of concentration; hence, a data analysis to quantify the NO_2_ concentrations in the software solution could be proposed using this system. It is also noticed in Figure S7b,c that the visualization time window in the App is one hour; therefore, even when the system can store more than 8 h, the data are visualized in 1 h segments for better visualization. This App is a custom-made Android application developed on the Android Studio platform, and it was developed for implementing these tests. The communication between the Android App and the system was conducted using a standard library that handles the Type 2 tag, MIFARE Ultralight protocol and initialization anticollision protocol activation, ISO 14443 A-3-2-1/ISO 18092, and NFC A tags [51].

## 4. Conclusions

This study demonstrates an effective technique to build a cumulative gas sensor using NFC technology. The integration of a sensing layer, the interdigitated electrodes, and the electronics on the same flexible printed circuit board gives a compact design, makes it easy to integrate with a working vest, and smooths the path to read out the sensor regardless of time and location. The synthesis of the zinc oxide nanoflowers and nanopillars mixed with graphene offered the possibility of obtaining sensors that work at room temperature. Also, it enabled obtaining sensors highly selective towards NO_2_ concentrations, having a sensitivity of 3% ppm^−1^ in 1 ppm level for both sensors (nanopillars and nanoflowers), and a theoretical LoD of 18.7 ppb (nanopillars) and 13.3 ppb (nanoflowers), a response of 20.24% ± 0.38% (nanoflowers) and 19.59% ± 0.24% (nanopillars) under 1 ppm exposure at 60% RH. The use of the airbrush technique facilitates the assembly process of the system without interfering with the electronic design.

The wearable NFC tag system proofreads the concept of being used in a target application, in which human resources could be exposed to NO_2_ concentrations during working hours. Nonetheless, a few improvements could be made to the final application. For instance, the inclusion of a visual or sonorous indicator when the target gas exposure increases would be suitable to alert the workers before reading the tag at the end of the working hours. Likewise, the inclusion of a humidity sensor would be suitable to address the effect of humidity on the sensor response with a multivariate analysis. Also, this system could be combined with standard alarm systems placed in the working area. Additionally, replacing the battery with a harvesting energy element would be an interesting approach to increasing the lifetime of this wearable NFC tag sensor.

It is important to notice that, to prove this concept, the selectivity toward NO_2_ of these sensors has been tested toward interfering species such as benzene, toluene, carbon monoxide, ethanol, ammonia, and hydrogen. Anyhow, the analysis of other possible interfering gases, such as ozone, would be suitable for the subsequent version of this study. Furthermore, we strongly believe that this work could be used with different sensing materials, and these results will provide a new perspective for on-site monitoring, recording, and analysis of threatening gases in working environments. Therefore, the use of new materials that guarantee faster response and recovery time at room temperature would be a suitable research topic to continue this work and be integrated with an improved version of this wearable NFC tag system.

## Data Availability

Data used in this paper is available on https://doi.org/10.5281/zenodo.10683822.

## Supplementary Materials

The following supporting information can be downloaded at: https://www.mdpi.com/article/10.3390/s24051431/s1, Table S1: Comparison of graphene-based gas sensors for NO_2_ detection at room temperature; Table S2: Comparison of wearable NFC gas sensors on a flexible substrate; Figure S1: Test bench of the power consumption measurement; Figure S2: FESEM image using Back-scattered electron detector of bare graphene; Figure S3: EDX of the nanoflowers sample; Figure S4: EDX of the nanopillars sample; Figure S5: EDX of the bare graphene; Figure S6: Raman spectroscopy of bare graphene; Figure S7: (a) Picture of the testbench for the test of the wearable system developed in this work; Screenshots of the Android App (b) capturing the exposure of 1 ppm NO_2_ (10 min), synthetic dry air (10 min), and 500 ppb (10 min); and (c) capturing the exposure of 500 ppb NO_2_ (10 min), synthetic dry air (10 min), and 1 ppb (10 min). References [52,53,54,55,56,57,58,59,60,61,62,63,64,65,66,67] are cited in the Supplementary Materials.
