# Supplementary material for: ZnO Decorated Graphene-Based NFC Tag for Personal NO2 Exposure Monitoring during a Workday
Source: Sensors (Basel). 2024 Feb 22;24(5):1431. doi: 10.3390/s24051431 (PMC10935135; doi:10.3390/s24051431)
Supplement: Supplementary file 1 [file sensors-24-01431-s001.zip › sensors-2835680-supplementary.pdf]

Supplementary materials

ZnO decorated Graphene-based NFC tag for personal NO_2 exposure monitoring during a workday

Alejandro Santos Betancourt [1], José C. Santos-Ceballos [1], Mohamed Ayoub Alouani [1], Shuja Bashir Malik [1], Alfonso J. Romero [1], José L. Ramírez [1], Xavier Vilanova [1*] and Eduard Llobet [1]

1- Universitat Rovira i Virgili, MINOS, Països Catalans, 26 Tarragona, Catalunya 43007, Spain;

* xavier.vilanova@urv.cat

Table S1: Comparison of graphene-based gas sensors for NO_2 detection at room temperature.

Material	Concentration (ppm)	Response %	Response Time (seconds)	Recovery Time (seconds)	Ref.
Co_3O_4-rGO	5	26.8	90	2400	[1]
rGO/ZnO-CT and ET	15	44	140	630	[2]
All graphene	5	12	328	1941	[3]
rGO/Ag nanowires	50	20	162	1200	[4]
Ag-S-RGO	50	75	12	20	[5]
RGO/Cu_2O	2	60	N/A	N/A	[6]
ZnS NPs/N-rGO	10	2.2	N/A	724	[7]
Graphene:ZnO	0.5	5.1	N/A	N/A	[8]
ZnO/SnO_2-rGO	5	141.0	32	92	[9]
ZnO/graphene aerogel	50	3.6	132	164	[10]
rGO/ZnO flowers and nanoparticles	1.5	1.4	394	807	[11]
rGO/ZnO laser modified	5	6.2	100	200	[12]
rGO/ZnO nanorods	100	17.4	780	1980	[13]
Graphene:ZnO NF	1	20.24	600	2820	This work
Graphene:ZnO NP	1	19.59	600	2940	This work

Table S2: Comparison of wearable NFC gas sensors on a flexible substrate.

Target gas	Active material	Sensing range	Sensitivity	Average Power consumption	Chip	Battery	Substrate	Weight	Ref
NH_3	Ag - rGO	5–100 ppm	1.25 %	N/A	no	no	PET	N/A	[14]
DCP	BMIMCl/HFIPN/ SWCNT	0.028–2.4 ppm	N/A	N/A	no	no	PET	N/A	[15]
NH_3	Cellulose fiber	0.2–1000 ppm	N/A	N/A	yes	no	Cellulose paper	N/A	[16]
NH_3	PTS–PANi	5–200 ppm	45 % ppm^{-1}	N/A	no	no	PET	N/A	[17]
Cadaverine	PTS–PANi	5 – 40 ppm	4.25 % ppm^{-1}	N/A	no	no	PET	N/A	[17]
Putrescine	PTS–PANi	5 – 40 ppm	9.2 % ppm^{-1}	N/A	no	no	PET	N/A	[17]
O_2	P4VP–SWCNTs–FeII	2 – 21 %	N/A	N/A	no	no	PET	N/A	[18]
CO_2	La_2O_2S:Eu/N/TMAOH/	up to 50,000 ppm	N/A	4.45 mW	yes	no	PET	N/A	[19]
Ethylene	SnO_2	100 – 500 ppm	N/A	N/A	yes	no	PET	N/A	[20]
O2, CO2; NH3	*	0.05 – 2 %, < 60 %, tens of ppm	N/A	8.5 mW	yes	no	PEN	N/A	[21]
NO_2	Graphene: ZnO	Up to 1 ppm	3 % ppm^{-1}	24.9 µW	yes	yes	PI	0.706 grams **	This work

* See reference for details

** The weight of the battery should be added to this quantity. (Ex: using a CR2032 battery, the total weight is around 4.5 grams)

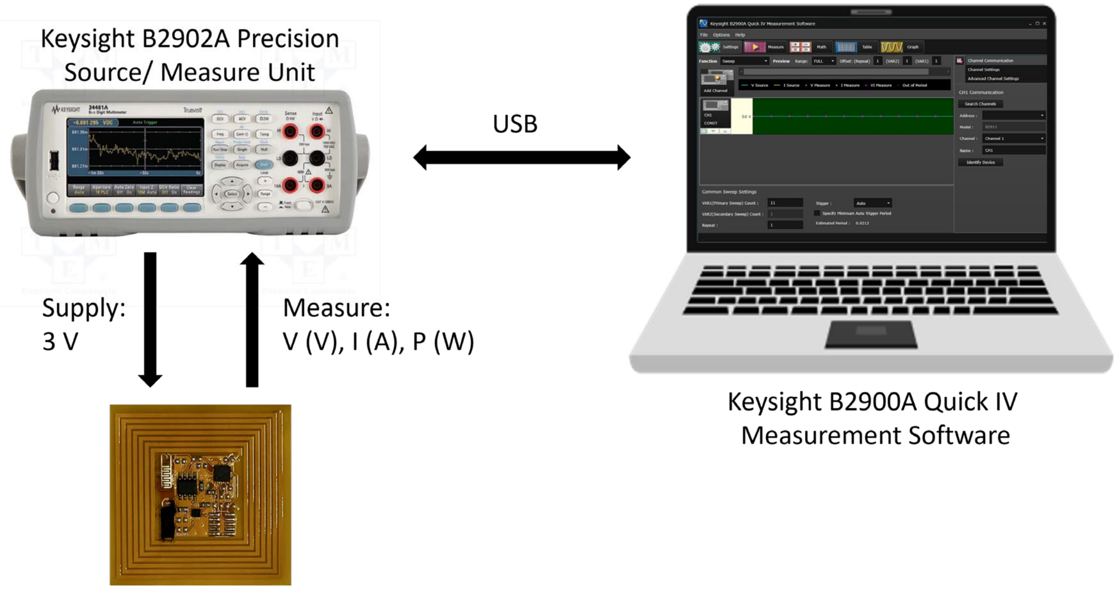

Figure S1: Test bench of the power consumption measurement.

The power consumption of the wearable NFC tag system was measured using Keysight B2902A Precision Source/ Measure Unit. The wearable was sourced with 3 V (according to the power

source specification in the electronic design) and the level of voltage, current, and power were acquired using the Software Quick IV Measurement version 4.1.1821.368 from Keysight Technologies. Ten-minute tests were conducted several times observing the system commuting between power modes (Deep Power Down Mode and RTC Wake Up). Similarly, the tests were repeated but reading a few times the data from the wearable with a smartphone running the test Android application. The resultant data was exported to MATLAB R2022b, plotted, and analyzed. As a result, the average power consumption in each section was: around 1.5 mW when reading the sensors every 60 seconds, around 1.25 mW when the device interacts with the NFC field to send the data from the sensor to the NFC reader, and around 3 µW when the device is in DPDM. This ultra-low power consumption value is consistent with the values reported by the manufacturer of the SoC NHS3152 and the DC-DC NCP705 in off mode.

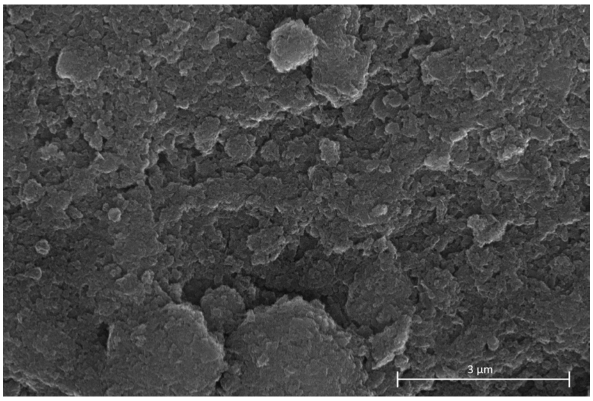

Figure S2: FESEM image using Back-scattered electron detector of bare graphene.

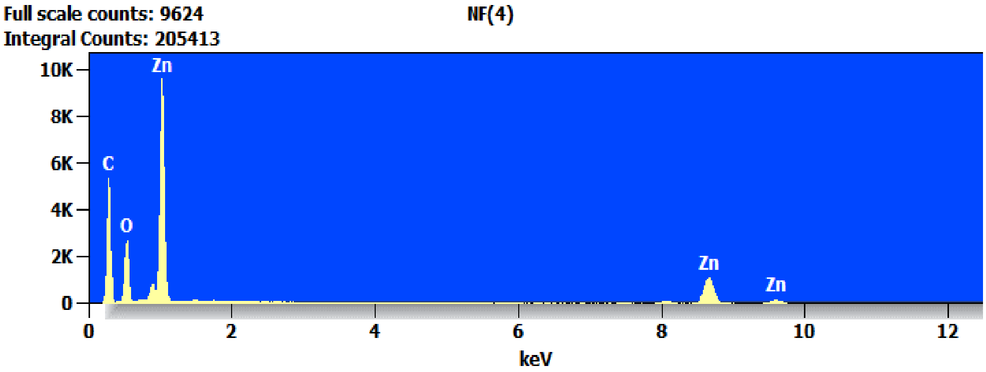

Figure S3: EDX of the nanoflowers sample

Live Time: 40.0 sec.

Wed Nov 29 14:35:01 2023

Filter Fit   Chi Squared:22.666

Errors: +/- 1 Sigma

Correction Method: Proza (Phi-Rho-Z)

Acc.Voltage: 20.0 kV Take Off Angle: 35.0 deg.

Quantitative Results for: NF(4)

Element	Weight %	Weight % Error	Norm. Wt.%	Atom %	Formula
C	38.29	± 0.22	38.29	58.55	C
O	27.82	± 0.29	27.82	31.94	O
Zn	33.89	± 0.46	33.89	9.52	Zn
Total	100.00		100.00	100.00	

A higher amount of Zn can be seen because the EDX was performed on top of the nanoflower. Details are indicated in its corresponding FESEM image (Figure 4 (b) of the principal document).

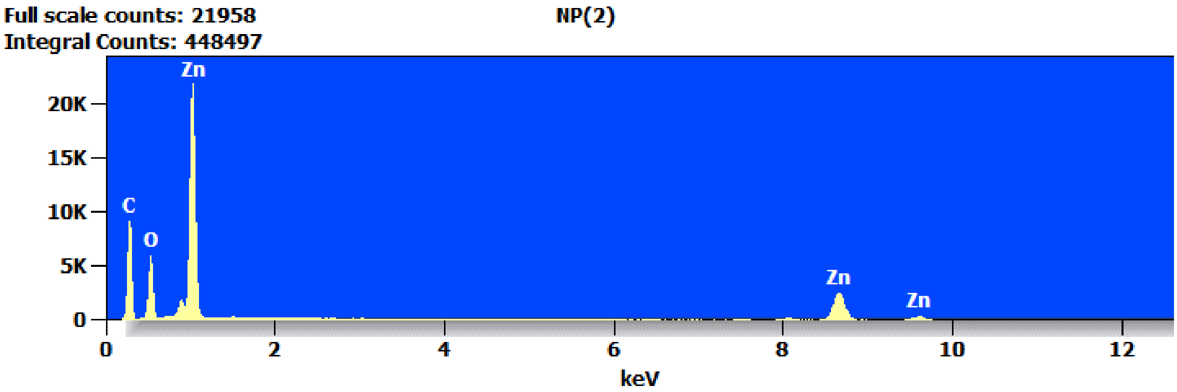

Figure S4: EDX of the nanopillars sample

Live Time: 40.0 sec.

Wed Nov 29 14:49:51 2023

Filter Fit   Chi Squared:43.982

Errors: +/- 1 Sigma

Correction Method: Proza (Phi-Rho-Z)

Acc.Voltage: 20.0 kV Take Off Angle: 34.4 deg.

Quantitative Results for: NP(2)

Element	Weight %	Weight % Error	Norm. Wt.%	Atom %	Formula
C	34.66	± 0.18	34.66	55.17	C
O	28.50	± 0.22	28.50	34.05	O
Zn	36.84	± 0.33	36.84	10.77	Zn
Total	100.00		100.00	100.00	

A higher amount of Zn can be seen because the EDX was performed on top of the nanopillars. Details are indicated in its corresponding FESEM image (Figure 4 (a) of the principal document).

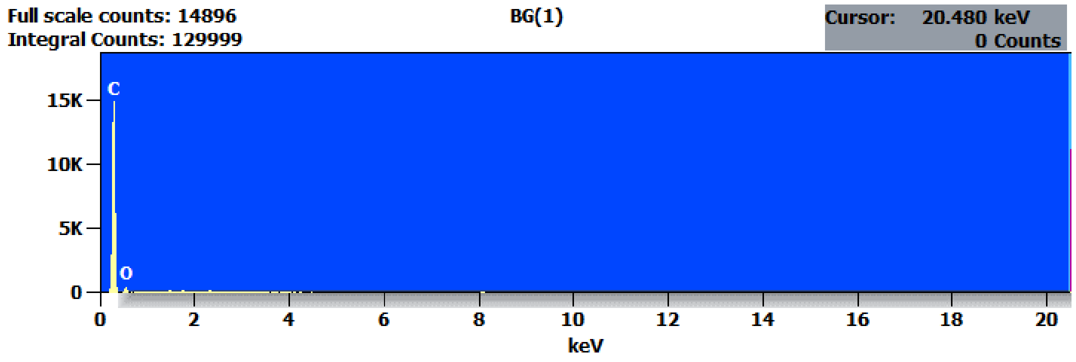

Figure S5: EDX of the bare graphene.

Live Time: 40.0 sec.

Wed Nov 29 14:59:29 2023

Filter Fit   Chi Squared:330.665

Errors: +/- 1 Sigma

Correction Method: Proza (Phi-Rho-Z)

Acc.Voltage: 20.0 kV Take Off Angle: 35.8 deg.

Quantitative Results for: BG(1)

Element	*Weight %*	*Weight % Error*	*Norm. Wt.%*	*Atom %*	*Formula*
C	80.84	± 0.39	80.84	84.89	C
O	19.16	± 0.54	19.16	15.11	O
Total	100.00		100.00	100.00	

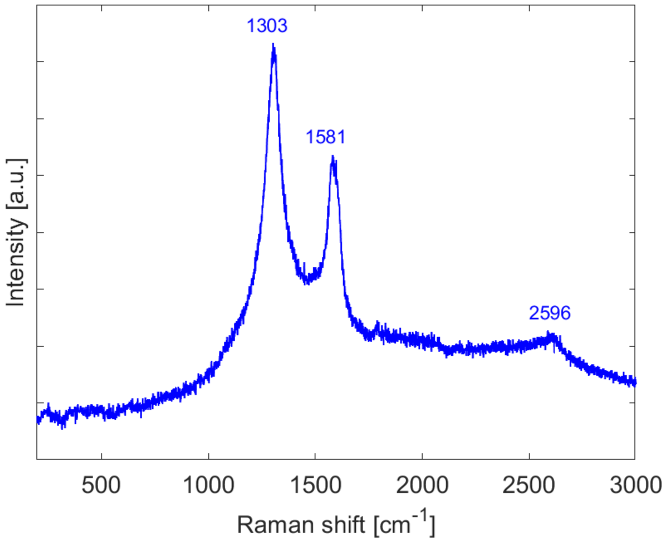

Figure S6: Raman spectroscopy of bare graphene.

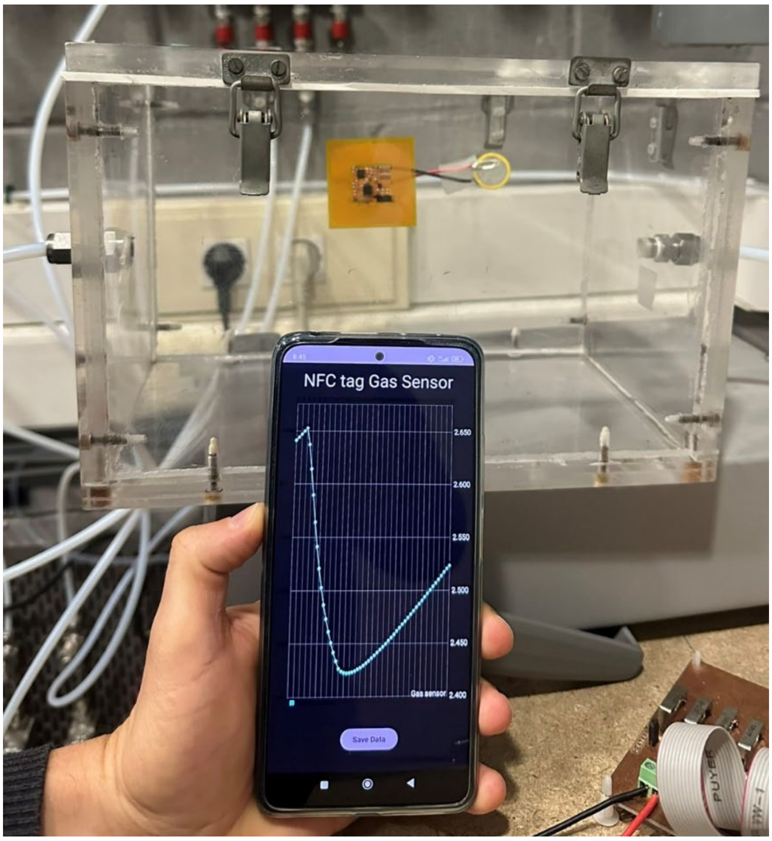

(a)

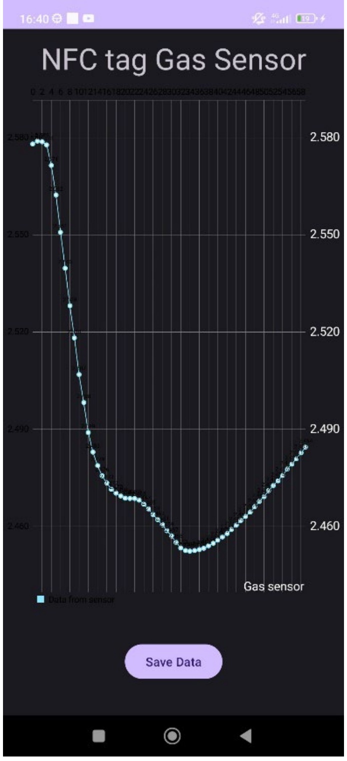

(b)

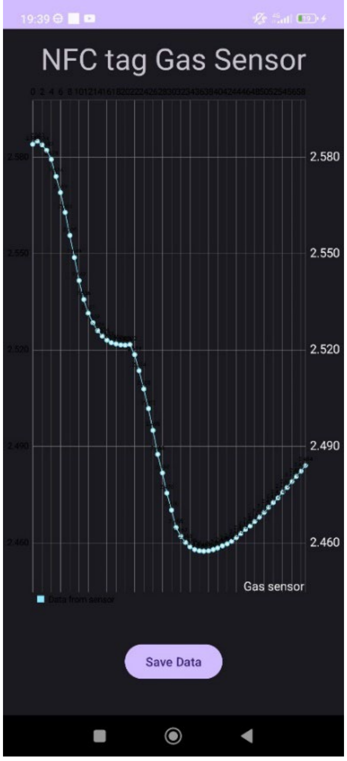

(c)

Figure S7: (a) Picture of the testbench for the test of the wearable system developed in this work; Screenshots of the Android App (b) capturing the exposure of 1 ppm NO_2 (10 minutes), synthetic dry air (10 minutes), and 500 ppb (10 minutes); and (c) capturing the exposure of 500 ppb NO_2 (10 minutes), synthetic dry air (10 minutes), and 1 ppb (10 minutes).

References

1. Zhang, B.; Cheng, M.; Liu, G.; Gao, Y.; Zhao, L.; Li, S.; Wang, Y.; Liu, F.; Liang, X.; Zhang, T.; et al. Room Temperature NO_2 Gas Sensor Based on Porous Co_3O_4 Slices/Reduced Graphene Oxide Hybrid. *Sens Actuators B Chem* **2018**, *263*, 387–399, doi:10.1016/j.snb.2018.02.117.

2. Li, W.; Chen, R.; Qi, W.; Cai, L.; Sun, Y.; Sun, M.; Li, C.; Yang, X.; Xiang, L.; Xie, D.; et al. Reduced Graphene Oxide/Mesoporous ZnO NSs Hybrid Fibers for Flexible, Stretchable, Twisted, and Wearable NO_2 E-Textile Gas Sensor. *ACS Sens* **2019**, *4*, 2809–2818, doi:10.1021/acssensors.9b01509.

3. Kim, Y.H.; Kim, S.J.; Kim, Y.-J.; Shim, Y.-S.; Kim, S.Y.; Hong, B.H.; Jang, H.W. Self-Activated Transparent All-Graphene Gas Sensor with Endurance to Humidity and Mechanical Bending. *ACS Nano* **2015**, *9*, 10453–10460, doi:10.1021/acsnano.5b04680.

4. Luan, Y.; Zhang, S.; Nguyen, T.H.; Yang, W.; Noh, J.S. Polyurethane Sponges Decorated with Reduced Graphene Oxide and Silver Nanowires for Highly Stretchable Gas Sensors. *Sens Actuators B Chem* **2018**, *265*, 609–616, doi:10.1016/J.SNB.2018.03.114.

5. Huang, L.; Wang, Z.; Zhang, J.; Pu, J.; Lin, Y.; Xu, S.; Shen, L.; Chen, Q.; Shi, W. Fully Printed, Rapid-Response Sensors Based on Chemically Modified Graphene for Detecting NO_2 at Room Temperature. *ACS Appl Mater Interfaces* **2014**, *6*, 7426–7433, doi:10.1021/am500843p.

6. Deng, S.; Tjoa, V.; Fan, H.M.; Tan, H.R.; Sayle, D.C.; Olivo, M.; Mhaisalkar, S.; Wei, J.; Sow, C.H. Reduced Graphene Oxide Conjugated Cu_2O Nanowire Mesocrystals for High-Performance NO_2 Gas Sensor. *J Am Chem Soc* **2012**, *134*, 4905–4917, doi:10.1021/ja211683m.

7. Chen, X.; Wang, T.; Han, Y.; Lv, W.; Li, B.; Su, C.; Zeng, M.; Yang, J.; Hu, N.; Su, Y.; et al. Wearable NO_2 Sensing and Wireless Application Based on ZnS Nanoparticles/Nitrogen-Doped Reduced Graphene Oxide. *Sens Actuators B Chem* **2021**, *345*, 130423, doi:10.1016/J.SNB.2021.130423.

8. Alouani, M.A.; Casanova-Cháfer, J.; Güell, F.; Peña-Martín, E.; Ruiz-Martínez-Alcocer, S.; de Bernardi-Martín, S.; García-Gómez, A.; Vilanova, X.; Llobet, E. ZnO-Loaded Graphene for NO_2 Gas Sensing. *Sensors* **2023**, *23*, 6055, doi:10.3390/S23136055/S1.

9. Wang, Z.; Gao, S.; Fei, T.; Liu, S.; Zhang, T. Construction of ZnO/SnO_2 Heterostructure on Reduced Graphene Oxide for Enhanced Nitrogen Dioxide Sensitive Performances at Room Temperature. *ACS Sens* **2019**, *4*, 2048–2057, doi:10.1021/acssensors.9b00648.

10. Liu, X.; Sun, J.; Zhang, X. Novel 3D Graphene Aerogel–ZnO Composites as Efficient Detection for NO_2 at Room Temperature. *Sens Actuators B Chem* **2015**, *211*, 220–226, doi:10.1016/J.SNB.2015.01.083.

11. Ugale, A.D.; Umarji, G.G.; Jung, S.H.; Deshpande, N.G.; Lee, W.; Cho, H.K.; Yoo, J.B. ZnO Decorated Flexible and Strong Graphene Fibers for Sensing NO_2 and H_2S at Room Temperature. *Sens Actuators B Chem* **2020**, *308*, 127690, doi:10.1016/J.SNB.2020.127690.

12. Lin, C.S.; Hsieh, H.F.; Ding, C.F.; Li, K.M.; Young, H.T.; Hsiao, W.T. Laser Surface Modification on RGO/ZnO Composite Materials for NO_2 Gas Sensing. *Mater Chem Phys* **2022**, *290*, 126551, doi:10.1016/J.MATCHEMPHYS.2022.126551.

13. Li, J.; Liu, X.; Sun, J. One Step Solvothermal Synthesis of Urchin-like ZnO Nanorods/Graphene Hollow Spheres and Their NO_2 Gas Sensing Properties. *Ceram Int* **2016**, *42*, 2085–2090, doi:10.1016/J.CERAMINT.2015.09.134.

14. Zhang, L.; Tan, Q.; Kou, H.; Wu, D.; Zhang, W.; Xiong, J. Highly Sensitive NH_3 Wireless Sensor Based on Ag-RGO Composite Operated at Room-Temperature. *Scientific Reports 2019 9:1* **2019**, *9*, 1–10, doi:10.1038/s41598-019-46213-9.

15. Zhu, R.; Azzarelli, J.M.; Swager, T.M. Wireless Hazard Badges to Detect Nerve-Agent Simulants. *Angewandte Chemie International Edition* **2016**, *55*, 9662–9666, doi:10.1002/ANIE.201604431.

16. Barandun, G.; Soprani, M.; Naficy, S.; Grell, M.; Kasimatis, M.; Chiu, K.L.; Ponzoni, A.; Güder, F. Cellulose Fibers Enable Near-Zero-Cost Electrical Sensing of Water-Soluble Gases. *ACS Sens* **2019**, *4*, 1662–1669, doi:10.1021/acssensors.9b00555.

17. Ma, Z.; Chen, P.; Cheng, W.; Yan, K.; Pan, L.; Shi, Y.; Yu, G. Highly Sensitive, Printable Nanostructured Conductive Polymer Wireless Sensor for Food Spoilage Detection. *Nano Lett* **2018**, *18*, 4570–4575, doi:10.1021/acs.nanolett.8b01825.

18. Zhu, R.; Desroches, M.; Yoon, B.; Swager, T.M. Wireless Oxygen Sensors Enabled by Fe(II)-Polymer Wrapped Carbon Nanotubes. *ACS Sens* **2017**, *2*, 1044–1050, doi:10.1021/acssensors.7b00327.

19. Escobedo, P.; Fernández-Ramos, M.D.; López-Ruiz, N.; Moyano-Rodríguez, O.; Martínez-Olmos, A.; Pérez de Vargas-Sansalvador, I.M.; Carvajal, M.A.; Capitán-Vallvey, L.F.; Palma, A.J. Smart Facemask for Wireless CO_2 Monitoring. *Nature Communications 2022 13:1* **2022**, *13*, 1–12, doi:10.1038/s41467-021-27733-3.

20. Choi, J.; Visagie, I.; Chen, Y.; Abbel, R.; Parker, K. NFC-Enabled Dual-Channel Flexible Printed Sensor Tag. *Sensors 2023, Vol. 23, Page 6765* **2023**, *23*, 6765, doi:10.3390/S23156765.

21. Escobedo, P.; Erenas, M.M.; López-Ruiz, N.; Carvajal, M.A.; Gonzalez-Chocano, S.; de Orbe-Payá, I.; Capitán-Valley, L.F.; Palma, A.J.; Martínez-Olmos, A. Flexible Passive near Field Communication Tag for Multigas Sensing. *Anal Chem* **2017**, *89*, 1697–1703, doi:10.1021/acs.analchem.6b03901.
